# Physicochemical Characterization and Biological Activities of Black and White Garlic: In Vivo and In Vitro Assays †

**DOI:** 10.3390/foods8060220

**Published:** 2019-06-21

**Authors:** María Ángeles Toledano Medina, Tania Merinas-Amo, Zahira Fernández-Bedmar, Rafael Font, Mercedes del Río-Celestino, Jesús Pérez-Aparicio, Alicia Moreno-Ortega, Ángeles Alonso-Moraga, Rafael Moreno-Rojas

**Affiliations:** 1 Department of Food Science and Health, IFAPA-Palma del Río, Avda. Rodríguez de la Fuente, s/n, 14700 Palma del Río, Córdoba, Spain; mariaa.toledano@juntadeandalucia.es (M.A.T.M.); jesus.perez.aparicio@juntadeandalucia.es (J.P.-A.); 2 Department of Genetics, University of Córdoba, Gregor Mendel Building, Campus Rabanales, 14071 Córdoba, Spain; tania.meram@gmail.com (T.M.-A.); ge1almoa@uco.es (A.A.-M.); 3 Agrifood Laboratory, CAPDER Avda. Menéndez Pidal s/n, 14004 Córdoba, Spain; rafaelm.font@juntadeandalucia.es (R.F.); mercedes.rio.celestino@juntadeandalucia.es (M.d.R.-C.); 4 Department of Bromatology and Food Technology, University of Córdoba, Darwin Building, Campus Rabanales, 14071 Córdoba, Spain; aliciamorenoortega@hotmail.com (A.M.-O.); rafael.moreno@uco.es (R.M.-R.); 5 Department of Food Science and Health, IFAPA-Alameda del Obispo, Avda. Menéndez-Pidal, s/n, 14004 Córdoba, Spain

**Keywords:** black garlic, physicochemical profile, polyphenol content, HL-60 cell line

## Abstract

White and three types of black garlic (13, 32, and 45 days of aging, named 0C1, 1C2, and 2C1, respectively) were selected to study possible differences in their nutraceutic potential. For this purpose, garlic were physicochemically characterized (Brix, pH, aW, L, polyphenol, and antioxidant capacity), and both in vivo and in vitro assays were carried out. Black garlic samples showed higher polyphenol content and antioxidant capacity than the white ones. The biological assays showed that none of the samples (neither raw nor black garlic) produced toxic effects in the *Drosophila melanogaster* animal genetic model, nor exerted protective effects against H_2_O_2_, with the exception of the 0C1 black garlic. Moreover, only white garlic was genotoxic at the highest concentration. On the other hand, 0C1 black garlic was the most antigenotoxic substance. The in vivo longevity assays showed significant extension of lifespan at some concentrations of white and 0C1and 1C2 black garlic. The in vitro experiments showed that all of the garlic samples induced a decrease in leukemia cell growth. However, no type of garlic was able to induce proapoptotic internucleosomal DNA fragmentation. Taking into account the physicochemical and biological data, black garlic could be considered a potential functional food and used in the preventive treatment of age-related diseases. In addition, our findings could be relevant for black-garlic-processing agrifood companies, as the economical and timing costs can significantly be shortened from 45 to 13 days of aging.

## 1. Introduction

Garlic (*Allium sativum*) is probably one of the oldest known medicinal plants, used since ancient times to cure different human diseases. Garlic started taking part in humans’ daily diet in Ancient Egypt [1]. Several scientific researches and clinical trials have been conducted during the last decade to determine the effects of garlic consumption on human health. Garlic’s principal medicinal uses have focused on prevention and treatment of cardiovascular disease by lowering blood pressure and cholesterol, and, more recently, on its antimicrobial properties and as a preventive agent for cancer [2,3].

The physiological effects of garlic are due mainly to the presence of volatile sulfur compounds like thiosulfates, which give it its characteristic pungent aroma. Several recent studies have shown that these organosulfur compounds show anti-cancer, anti-cardiovascular, anti-neurological, and anti-liver disease effects, as well as effects for the prevention of allergies and arthritis [4,5,6,7]. This group of compounds, originating from the allicin decomposition, are associated with *Allium* species’ pungent aroma and taste as well as their antioxidant activity [4,8]. However, rats fed with fresh garlic at high doses (0.5 g/kg of body weight/day) showed toxicity in the liver [9].

Even though the health benefits of garlic are known, its global consumption is declining. In general, people are reluctant to eat raw garlic due to its pungent taste, smell, and gastrointestinal discomfort. Because of this, researchers are interested in developing aged garlic products to decrease these negative effects [10].

With a growing awareness of the health benefits of garlic, black garlic, an aged garlic product, has emerged as one of the fastest-growing health-oriented food products in world markets [10]. Black garlic is produced through the natural aging of whole ordinary garlic under controlled high temperature (70 °C) and humidity (90%) conditions for several days, without any artificial treatments or additives [11]. Thermal processes are commonly used in food manufacturing to enhance the sensorial quality of foods, their palatability, and to extend the range of colors, tastes, aromas, and textures in food [12]. In addition, heating processes have led to the formation of biological compounds that are not originally present in food [13]. However, the influence of thermal processes on the concentration of single flavonoids and phenolic acids in garlic still remains unknown.

During aging, the cloves of normal garlic change their color from white to brown and finally become black due to the Maillard reaction. At the same time, unstable compounds in raw garlic are transformed into stable soluble compounds with a high antioxidant power [6,14]; the organoleptic characteristics in black garlic are improved due to the conversion of unstable and odorous compounds to stable and odorless compounds such as S-allyl-L-cysteine (SAC), or decomposed to organosulfur compounds such as diallyl sulphide (DAS), diallyl disulphide (DADS), diallyl trisulphide (DATS), dithiins, and ajoene [4,6]. Previous studies on black garlic have reported that the increase in its antioxidant capacity could be due to the increase in polyphenols and S-allyl-cysteine, a compound derived from alliin, during the heat processing [15].

Compared with fresh garlic, black garlic contains a polyphenol content that is three times higher in whole black garlic bulbs and six times higher in peeled black garlic cloves [11], which is directly related to the increase in the antioxidant activity. The amino acids, carbohydrates, and the S-allyl-L-cysteine contents are increased 2.5 times, 28.7%–47.0%, and eight times, respectively [16,17].

Different beneficial health properties of black garlic have been described previously: (i) antioxidant effects using different indicators such as super-oxide dismutase (SOD), 2,2′-azino-bis(3-ethylbenz-thiazoline-6-sulfonic acid) (ABTS), and hydroxy radical scavenging, as well as Fe^2+^-chelating activities; (ii) in vivo and in vitro chemopreventive effects in different cancers—including ethanol extracts of aged black garlic, which reduce the viability of several human cancer cell lines (i.e., AGS, A549 lung, HepG2 liver, and MCF-7 breast cancer cells), and hexane extracts, which induce caspase-dependent apoptosis in leukemic cells; (iii) anti-inflammatory effects have been shown by inactivation of NF-κB, upregulation of heme oxygenase-1, and inhibition of the COX-2 and 5-lipooxygenase activities, among other effects [10].

The aim of the present study is to perform a qualitative and quantitative evaluation of the health-beneficial activities of white and three types of black garlic using a multi-assay experimental design at the individual, cell, and DNA levels. We assessed the genotoxic, antigenotoxic, and lifespan effects using an in vivo animal model of the common fruit fly (*Drosophila melanogaster*) and their proapoptotic capacities against cancer processes, including cytotoxicity and clastogenic DNA activity, using an in vitro human cancer model (HL-60 cell line). 

## 2. Materials and Methods 

### 2.1. Preparation of Samples

White and black garlic were used in this study. Raw white garlic was purchased in a local market. Black garlic was manufactured at 60 °C and 90% relative humidity (RH). Samples at 0 (White), 13 (0C1), 32 (1C2), and 45 (2C1) days aging were taken during the manufacturing process. After peeling bulbs, samples were crushed and divided into three subsets (to be physicochemically analyzed at specified times). Garlic samples were lyophilized (−20 °C, less than 1% water) before the biological assays and then dissolved in distilled water to obtain the different concentrations tested. The lyophilized extracts were stored at room temperature in a dark and dry atmosphere until use. 

The concentrations of garlic for the different bioassays were established taking into account the average daily food intake of *D. melanogaster* (1 mg/day) and the average body weight (1 mg) [18]. The concentration range for all tested substances was calculated in order to make it comparable to the recommended garlic daily intake for humans. Although there is no standard intake for garlic, the German Kommission E monograph proposed that a daily intake of approximately 1–2 garlic cloves (about 4 g) of intact garlic may have health benefits [19]. Unfortunately, this recommendation is not substantiated by any scientific reference.

### 2.2. Measurement of Soluble Solid Content, pH, a_w_, and Browning Intensity

Total soluble solid content (°Brix), pH, water activity (*a_w_*), and browning intensity (*L* value) values were determined in triplicate for all samples following the method previously described by Toledano–Medina et al. [11]. Garlic soluble solids (°Brix) were measured with an Abbe Refractometer ORT-1 of KERN (Kern & Sohn GmbH, Balingen-Frommern, Germany). Garlic pH was measured with a pH meter Crison Basic 20 (Crison Instruments, Barcelona, Spain). Garlic water activity (*a_w_*) was measured with an Aqualab Series 3/3TE meter with a temperature stabilizer (MeterGroup, München, Germany). Garlic browning intensity was determined with a Konica Minolta CR-410 Croma Meter colorimeter (Konica Minolta, Inc., Tokyo, Japan) as an *L* value (*L* = 100, white; *L* = 0, black), following the method described previously by Toledano–Medina et al. [11].

### 2.3. Total Polyphenol Content and Antioxidant Capacity

A Perkin Elmer Lambda 20 UV VIS spectrophotometer (Perkin Elmer, Waltham, MA, USA) was used to determine total polyphenol content and antioxidant capacity in raw and heated garlic. A previous extract was prepared to analyze antioxidant properties. Briefly, samples were lyophilized (−20 °C, less than 1% water) and spliced into five extracts per sample. Garlic extract was prepared dissolving 0.3 g of the lyophilized sample in 10 mL of a mixture of 50% (*v/v*) ethanol and distilled water. Next, samples were stirred for one hour and then filtered using a Buchner funnel with Whatman paper (Whatman PLC, Maidstone, UK) into a vacuum flask connected to a vacuum pump filter. The filtered extract was levelled at 25 mL with a 50% (*v/v*) hydroalcoholic solution.

The polyphenol concentration of garlic samples was determined by the Folin–Ciocalteu method [20]. To a volumetric 25 mL flask, 0.5 mL of extract, 10 mL of distilled water, 1 mL of Folin–Ciocalteu reagent, and 3 mL of sodium carbonate 20% (*w/v*) were added and diluted to volume (25 mL) with distilled water. The mixture was heated to 50 °C for 5 min to accelerate the coloration reaction. Subsequently, it was cooled with water, and the reading was carried out in the spectrophotometer (Perkin Elmer) at 765 nm. The reading was compared with a calibration curve prepared with different gallic acid solutions: 75, 100, 200, 250, 300 ppm. Polyphenol content results were expressed considering the dilution of the sample (0.3 g in 25 mL) in grams of gallic acid equivalents per kilogram of lyophilized sample.

Raw and heated garlic antioxidant capacity was determined by the ABTS method [21]. A mix of 2.557 mL of a solution of 7 mM ABTS reagent (Sigma, St. Louis, MI, USA) and 0.333 mL of a solution of 2.25 mM potassium persulfate in distilled water was made. This solution was stored in darkness for 16 h, enough time for radical cation (ABTS^•+^) formation. Then, 0.15 mL of the ABTS^+^ solution was diluted in 15 mL of ethanol. The absorbance value at 734 nm was adjusted near 0.7 (A_0_). Next, 0.980 mL of ABTS^+^ solution and 0.02 mL of garlic extract were added. After stirring, the absorbance was read at 734 nm after 7 minutes (A_1_). The inhibition percentage was calculated by the following expression:

% inhibition = (A_0_ − A_1_) × 100/A_0_. (1)

A calibration curve was built with the following Trolox (6-hydroxy-2, 5, 7, 8-tetramethylchroman-2-carboxylic acid) concentrations: 0.1, 0.5, 1, and 1.5 mM. Considering the sample dilution, results were expressed in mmol Trolox-equivalents per kilogram of lyophilized sample.

### 2.4. In Vivo Assays

#### 2.4.1. *D. melanogaster* Strains

The following *Drosophila* strains, each carrying a third chromosome hair marker, were used: (i) *mwh/mwh* are homozygous for the recessive multiple wing hairs (*mwh*) mutation that produces multiple tricomas per cell instead of one [22], and (ii) *flr^3^/In (3LR) TM3, rip^p^sep bx^34e^e^s^Bd^S^*, where the *flr^3^* (*flare*) marker is a homozygous recessive lethal mutation that produces deformed tricomas but is viable in homozygous somatic cells once larvae start the development [23]. For detailed information on the mutations, see Lindsley and Zimm [24].

#### 2.4.2. Toxicity and Antitoxicity Assays

Five concentrations (4, 2, 1, 0.5, and 0.25 mg/mL) for each tested garlic, along with negative (H_2_O) and positive (0.12 M H_2_O_2_) controls were assayed after toxicity screening experiments. The toxicity index was calculated as the percentage of individuals born in each treatment with respect to the negative control. The antitoxicity tests consisted of combined treatments using the same concentrations as in the toxicity assays, with the exception of the highest one (4 mg/mL), by adding the toxicant hydrogen peroxide at 0.12 M [25]. The percentage of emerging adults was compared with the positive control. 

#### 2.4.3. Genotoxicity and Antigenotoxicity Assays

The genotoxicity assays were carried out following the method described by Graf et al. [26]. Briefly, trans-heterozygous larvae for *mwh* and *flr^3^* gene markers were obtained by crossing four-day-old virgin *flr^3^* females with *mwh* males in a 2:1 ratio. Four days after fertilization, females were allowed to lay eggs in fresh yeast medium (25 g yeast and 4 mL sterile distilled water) during 8 h to obtain synchronized larvae. After 72 ± 4 h, the larvae were collected, washed with distilled water to remove the remaining medium, and transferred, in groups of 100 individuals, to the treatment tubes where they were chronically fed with the different compounds. Treatment tubes contained 0.85 g of *Drosophila* Instant Medium (Formula 4-24, Carolina Biological Supply, Burlington, NC, USA) and 4 mL of solutions with different concentrations of garlic (2 mg/mL and 0.25 mg/mL).

The antigenotoxicity trials were carried out following the method described by Graf et al. [27], which consists of combined treatments of genotoxin (0.12 M H_2_O_2_) (Sigma, cat. number H-1009) and the same concentrations used in genotoxicity assays of lyophilized garlic. For the evaluation of the inhibition potency, negative (H_2_O) and positive (0.12 M H_2_O_2_) (Sigma, cat. number H-1009) concurrent controls were included. After emergence, adult flies were stored in 70% ethanol until the removal and mounting of wings on slides using Faure’s solution (30 g Arabic gum, 20 mL glycerol, 50 g chloral hydrate, and 50 mL distilled water) for mutation screening under a photonic microscope (Leica, Wetzlar, Germany) at 400× magnification.

Similar numbers of male and female wings for each treatment and concentration were mounted, and wing hair mutations were scored among a total of 24,400 monotricoma wild-type cells per wing [28].

Wing hair spots were grouped into three different categories: *S*, a small single spot corresponding to one or two cell clones exhibiting the *mwh* phenotype that occurs in the latest stages of the mitotic division; *L*, a large single spot with three or more cell clones showing *mwh* or *flr*^3^ phenotypes that occur in the early stages of larval development; or *T*, a twin spot corresponding to two juxtapositioned clones, one showing the *mwh* phenotype and other the *flr^3^* phenotype. Small and large spots are caused by somatic point mutations, chromosome aberrations, and somatic recombinations, while twin spots are produced exclusively by somatic recombinations between the *flr^3^*locus and the centromere. 

The total number of clones was also counted and a multiple-decision procedure was applied to determine whether a result was positive, inconclusive, or negative [29,30]. The inhibition percentages (IPs) for the combined treatments were calculated from the total spots per wing statistics with the following formula [31]:

IP = ((single genotoxin − combined treatment)/single genotoxin) × 100.(2)

#### 2.4.4. Lifespan Assays

In order to compare the genotoxicity and longevity results, flies that underwent the lifespan trials carried the same genotype as in genotoxicity assays. Hence, the F1 progeny from *mwh* and *flr^3^* parental strains produced by a 24 h egg-laying in fresh yeast medium was used in the longevity experiments. All experiments were carried out at 25 °C and according to the procedure described by Fernández–Bedmar et al. [25]. Briefly, synchronized 72 ± 12 hour-old trans-heterozygous larvae were washed, collected, and transferred in groups of 100 individuals to test vials containing 0.85 g of *Drosophila* Instant Medium (formula 4-24, Carolina BiologicalSupply, Burlington NC, USA) and 4 mL of the different concentrations of the selected compounds.

Sets of 25 emerged individuals of the same sex were selected and placed into sterile vials containing 0.21 g of *Drosophila* Instant Medium (formula 4-24, Carolina BiologicalSupply, Burlington NC, USA) and 1 mL of the different concentrations of solution of the compounds (4 mg/mL–0.25 mg/mL range). Two replicates were followed during the complete life extension for each control and for the concentrations established. Alive animals were counted, and the media was renewed twice a week.

### 2.5. In Vitro Assays

#### 2.5.1. HL-60 Cell Line Culture Conditions

Cells were grown in RPMI-1640 medium (Sigma, R5886, St. Louis, MI, USA) supplemented with 50 mL heat-inactivated fetal bovine serum (Linus, S01805, Madrid, Spain), L-glutamine at 200 mM (Sigma, G7513), and antibiotic-antimycotic solution with 10,000 units of penicillin, 10 mg of streptomycin, and 25 μg amphotericin B per mL (Sigma, A5955). Cells were incubated at 37 °C in a humidified atmosphere of 5% CO_2_ (Shel Lab, Cornelius, OR, USA) [32]. The cultures were plated at 2.5 × 10^4^ cells/mL density in 10 mL culture bottles and passed every 2 days.

#### 2.5.2. Cytotoxicity Assay

HL-60 cells were placed in 96-well culture plates (2 × 10^4^ cells/mL) and treated for 72 h with the lyophilized white and black garlic at different concentrations (4 mg/mL, 2 mg/mL, 1 mg/mL, 0.5 mg/mL, 0.25 mg/mL, 0.12 mg/mL, 0.06 mg/mL, 0.03 mg/mL, and 0.015 mg/mL for white garlic and 4 mg/mL, 2 mg/mL, 1 mg/mL, 0.5 mg/mL, and 0.25 mg/mL for black garlic samples). This wide range of concentrations was intended to estimate the inhibitory concentration 50 (IC_50_). 

Cell viability was determined by the trypan blue dye (Sigma, T8154) exclusion test. Trypan blue solution was added to the cell cultures at a 1:1 volume ratio and 20 μL of cell suspension were immediately loaded into a Neubauer chamber. Cells were counted with an inverted microscope at 100× magnification (AE30/31, Motic, Wetzlar, Germany). Curves were plotted as survival percentages with respect to the control growing at 72 h. At least three independent repetitions were carried out.

#### 2.5.3. Determination of DNA Fragmentation

DNA fragmentation is a hallmark of apoptosis and has been regarded as a critical step in apoptosis. Briefly, HL-60 cells (1 × 10^6^ cells/mL) were treated with different concentrations of lyophilized garlic (4 mg/mL, 2 mg/mL, 1 mg/mL, 0.5 mg/mL, and 0.25 mg/mL, respectively) for 5 h. Treated cells were collected and centrifuged at 3000 rpm for 5 min, and DNA was extracted with lysis, precipitation, and wash steps according to Merinas–Amo et al. [33]. The total extracted DNA was quantified in a spectrophotometer (Nanodrop^®^ ND-1000, Thermo Fisher Scientific, Waltham, MA, USA), and 1200 ng of DNA was loaded into a 2% agarose gel electrophoresis, stained with ethidium bromide, and visualized under UV light.

### 2.6. Statistical Analysis 

The statistical analysis of the solid content, pH, *a_w_*, browning intensity, polyphenol content, antioxidant capacity, and total polyphenol index for each type of garlic was evaluated with the SPSS Statistics 17.0 software SPSS (IBM, Armonk, NY, USA) using one-way ANOVA and Tukey’s test (homogeneous subsets) to assess the significance of the subsets.

Significant differences with respect to the concurrent control in toxicity assays were determined using the Chi-square method, and a concentration was considered as a toxic when the Chi-square value was higher than 5.02.

The frequency of each type of mutant clone/wing in the anti/genotoxicity assays was compared with the negative concurrent control, and significance was given at the 5% error level. Inconclusive and positive results were further analyzed with the Mann–Whitney–Wilcoxon (*α* = *β* = 0.05) nonparametric U-test using the SPSS Statistics 17.0 software SPSS.

The statistical treatment of life- and health-span data for each control and concentration was assessed with the SPSS Statistics 17.0 software, using the Kaplan–Meier method. The significance of the curves was determined using the Log-Rank method (Mantel-Cox).

To obtain the tumor growth inhibition curves, the mean of three independent assays of the alive-treated cells for each compound and concentration was used. The standard errors of the three repetitions were calculated, and the Excel-given curve was added. Finally, the inhibitory concentration 50 (IC_50_) was estimated.

## 3. Results and Discussion

### 3.1. Soluble Solids Content, pH, Water Activity, and Browning Intensity

A weight reduction was observed during the garlic manufacturing procedure, with the 0C1 black garlic being the sample with the nearest weight to the white garlic (Table 1). According to similar studies, changes in garlic weight during processing are mainly caused by a reduction of the amount of water [16]. The main organosulfur in black garlic is considered to be the water-soluble *S*-allyl-L-cysteine (SAC) [34]. Hence, after aging, SAC increased in the processed black garlic matrix, and its precursor garlicγ-glutamyl-*S*-allyl-L-cysteine decreased [10]. The manufacturing of black garlic in this manner is not a microbe-associated fermentation but a Maillard and Browning reaction because the processing temperature of garlic does not allow bacterial growth to elicit fermentation [16].

Soluble solids content (ºBrix), pH, water activity (*a_w_*), and browning intensity (*L*) are shown in Table 1. During heat treatment, soluble solids content increased in garlic, whereas pH, *a_w_*, and browning intensity decreased. Similar Tukey’s test values were obtained in °Brix readings for white and 0C1 black garlic (40.47), meanwhile, significant soluble solids content differences were observed in the 1C2 and 2C1 black garlic (43.17 and 45.67, respectively). The sugar content (°Brix) of black garlic increased with respect to white garlic. This result is in agreement with the data of Choi et al., which show that sugar content (e.g., glucose, fructose, sucrose, and maltose) increased in black garlic compared to fresh and steamed garlic [35]. Furthermore, this increment might be related to its sweeter taste [16]. pH significantly decreased during the manufacturing process. White garlic pH was the highest with a value of 5.94, whereas black garlic pH decreased rapidly starting at 3.69 and reaching 3.49 at 45 days of aging. These results are in agreement with the report by Shin et al., who showed that black garlic pH decreased from 6.40 to 5.29 after 6 days of aging [36]. The same observation has recently been described [11,37]. Water activity (*a_w_*) decreased with aging to a lesser extent than other parameters because the black garlic was manufactured maintaining a high relative humidity. According to Kaanane and Labuza and Labuza and Saltmarch, the rate of the browning reaction is known to reach a maximum at *a*_w_ values in the range of 0.5–0.7 [38,39]. However, significant differences between white and black garlic *a_w_* are found (Table 1). The high RH and time required for producing black garlic in the present study might have created a balanced situation between the *a*_w_ of the heated garlic sample and the RH inside the chamber where black garlic was produced. This *a*_w_ condition is thought to facilitate the browning reaction in heated garlic samples. As Table 1 shows, browning intensity (*L*) in white and black garlic was significantly different, with more than 28 units of difference between them, although the 1C2 and 2C1 black garlic showed similar luminescence (17.85 and 17.58, respectively). Browning intensity happened earlier at higher temperatures. Several studies have shown a positive relationship between temperature increasing and browning product formation; however, at the initial induction period a decrease is observed [40,41]. The garlic’s color eventually changed to dark brown/black, mainly due to the formation of numerous compounds resulting from the non-enzymatic browning reaction (Maillard reaction).

### 3.2. Total Polyphenol Content and Antioxidant Capacity

Total polyphenol (g/kg in Gallic) content and antioxidant capacity (inhibition percentage) are shown in Table 1. During heat treatment, unstable compounds of raw garlic are transformed into stable soluble compounds with a high antioxidant power [6,14]. Previous studies on black garlic reported that this enhancement of the antioxidant capacity could be due to the increase in polyphenols and *S*-allyl-cysteine, the compound derived from alliin [15]. The antioxidant power of polyphenols has been demonstrated, so it seems logical to state that an increase in polyphenol content in black garlic is responsible for the antioxidant properties in this product [42]. It is well known that the higher antioxidant effect of black garlic is due to the presence of *S*-allyl-cysteine, a compound derived from alliin during heat processing [15]. 

Significant differences among all the samples were found for the total polyphenol content and the antioxidant capacity. Both parameters increased significantly as heat increased. The highest concentration of polyphenol content was obtained in 2C1 black garlic, although all black garlic samples showed increases between 6 and 12 times in relation to the heat treatment (Table 1). Previous studies carried out with whole bulbs of black garlic at 70, 72, 75, and 78 °C have described an increase of 2–3 times in polyphenol content compared to raw garlic [11,43]. Our results on the increase of polyphenol content after heating agree in part with those obtained by other authors who found a threefold increase in content [43]. 

To clarify the antioxidant properties of black garlic during aging, we focused on the analysis of total polyphenol content. At the end of the heating process, an increase in antioxidant capacity was observed in garlic. Black garlic samples showed an increase rank of 5.7–7.8 times with respect to white garlic (Table 1). Several studies described that aged black garlic exerts stronger antioxidant activity than white garlic, both in vitro and in vivo assays [15,44]. The total polyphenol content of black garlic was not only significantly higher than that of raw garlic, but also increased significantly at the 13th day of aging. Similar results were obtained by Sasaki et al., who showed an antioxidant potency increase in aged black garlic extracts reaching 25-fold compared with fresh garlic [16]. According to Xu and Chang, heat treatment of the phenolic compounds increased the free fraction of phenolic acids, whereas it decreased the ester, glycoside, and ester-bound fractions, leading to an increase in free phenol forms [45]. Gorinstein et al. showed that the garlic processing conditions lead to changes in the content of its bioactive compounds (polyphenols such as flavonoids and anthocyanins), and this is related to the type and duration of treatment [46]. From the results regarding total polyphenols and antioxidant capacity, it is possible to state that the optimum aging period for black garlic in order to maximize antioxidant content may be 13 days.

### 3.3. Toxicity/Antitoxicity

**Figure 1:**
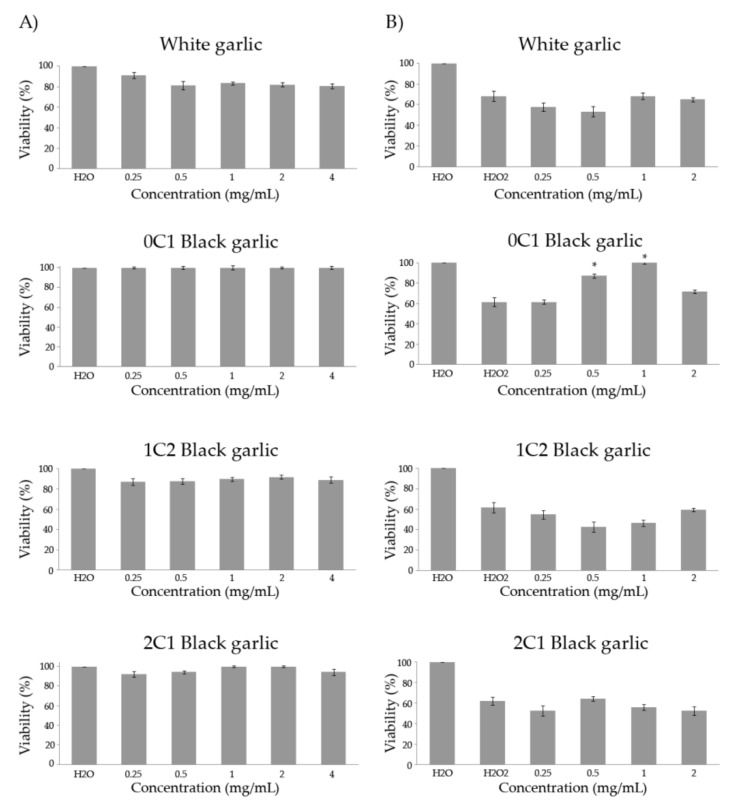
Toxicity (**A**) and antitoxicity (**B**) levels of black and white garlic studied in *D. melanogaster*. (**A**) Percentage of viability of *Drosophila* treated with different concentrations of the assayed garlic. (**B**) Viability of *Drosophila* tested with different concentrations of the tested garlic combined with the genotoxicant hydrogen peroxide at 0.12 M. Values represent the mean ± SE from three independent experiments. *: significant (*p* ≤ 0.05), with respect to their concurrent controls. 0C1: black garlic with 13 days aging, 1C2: black garlic with 32 days aging, and 2C1: black garlic with 45 days aging.

The toxicity and antitoxicity of the four samples of tested garlic was assessed in the *D. melanogaster* in vivo model. Figure 1A shows the relative percentage of emerging adults after treating larvae with different concentrations of these substances, showing that none of the garlic samples were toxic at the assayed doses in *D. melanogaster*. These results agree with those by which the safety of garlic extracts was well established through general, chronic, acute, and subacute toxicity, teratogenicity, and toxicity tests conducted by the U.S. Food and Drug Administration, and clinical studies as well [47,48,49,50,51,52].

Figure 1B shows the results of the antitoxicity assays using hydrogen peroxide as a toxicant. The individuals treated with 0.12 M of the oxidative toxin reached an average survival rate of 63.4% with respect to the water negative control. In addition, 0C1 black garlic was the only preventive substance against H_2_O_2_ at two of the assayed concentrations (0.5 and 1 mg/mL). On the other hand, white and 1C2 and 2C2 black garlic did not exhibit protective effects against the genotoxin at any tested concentrations (0.25–0.5 and 0.5–1 mg/mL, respectively). Lei et al. studied the effects that black 10–15 days-aged garlic extracts had in *D. melanogaster*. The results from this study demonstrated that black garlic extracts possess strong antioxidant capacities in vitro and in vivo [53].

### 3.4. Genotoxicity/Antigenotoxicity

To assess the genotoxicity/antigenotoxicity of the studied compounds, we used the SMART (Somatic Mutation and Recombination Test) Test in *D. melanogaster* [27]. Increasing concentrations of tested compounds, a negative control corresponding to water used as a solvent, and a positive control (H_2_O_2_) for periodic validation of the assay were concurrently assayed. Furthermore, antigenotoxicity experiments were carried out using combined treatments consisting of repeating every concentration tested and by adding the same concentration of hydrogen peroxide, which we have demonstrated to be a potent mutagen in the SMART system [54].

Table 2 shows the results of genotoxicity assays in the SMART test for white and the three black garlics. Negative controls showed a frequency of mutations per wing equal to 0.195, which falls into the historical range for the wing spot test [33,55]. The final concentration of H_2_O_2_ used (0.12 M) has been demonstrated to exert a potent genotoxic effect capable of inducing somatic mutations and mitotic recombination in *D. melanogaster* [56]. The average frequency of total mutations per wing obtained in the treatment with H_2_O_2_ was 0.425. For each concentration and compound, single small, single large, twin, and total clones were analyzed in the wings of chronically treated animals. The results showed that all garlic showed non-genotoxic activity except for the white one, which significantly increased the frequency of mutations to 0.425 at the highest concentration tested. Similar results were obtained by Abraham and Kesavan and Shukla and Taneja, who demonstrated that aqueous garlic extracts (5% *v/v*) and fine garlic powder (7.5, 5 and 2.5 g/kg body weight) supplementation do not induce chromosomal aberrations nor DNA damage in mouse bone marrow cells [57,58]. Similar results were obtained by Sowjanya et al. at 3, 6, and 12 mg/culture in human lymphocytes [59] and by Chughtai et al. using extracts of fresh garlic bulbs in a yeast model [60].

Vegetables contain polyphenols and oligoelements with antimutagenic activity [61]. The 0C1 black garlic was the only one able to inhibit the genotoxic activity of hydrogen peroxide in a dose-dependent manner (Table 2). The highest concentration tested for 0C1 black garlic in the combined treatments partially counteracted part of the genotoxic effect of H_2_O_2_, showing a decrease in the total mutation frequency to 0.266 spots/wing and inhibiting around 37% of the genotoxicity induced by H_2_O_2_ (without control correction). The rest of the compounds tested did not show significant protective results against DNA damage at the highest concentration and a just a slight inhibition percentage of mutations induced by the genotoxin were observed (24% for white, 18.6% for 1C2 black, and 7.5% for 2C1 black garlic). 

In general, garlic has significant antioxidant activity and protective effects against oxidative DNA damage regardless of the processing method [62]. Our antitoxicity and antigenotoxicity results showed that 0C1 black garlic (aged for 13 days) is able to protect from the genomic damage of this genotoxin in a dose-dependent manner. This effect could probably be due to the antioxidative and free-radical scavenging capacity of their respective organosulfur compounds, which agree with previous reports [14,63,64]. Besides the antioxidant activity, our results about the stronger antioxidant activity shown by black garlic, compared with fresh garlic, are in agreement with previous in vivo and in vitro garlic assays [65,66].

### 3.5. Longevity Assays 

*Drosophila melanogaster* is a choice model organism in the study of aging due to its relatively short life expectancy. Moreover, a large number of individuals can be reared in controlled laboratory conditions, and adults show many aspects of the observed cellular senescence events in mammals. Thus, flies have been frequently used to study physiological and pathological processes that affect life expectancy and can help to understand the relationship between nutrient metabolism and the mechanisms of aging [25]. 

**Figure 2:**
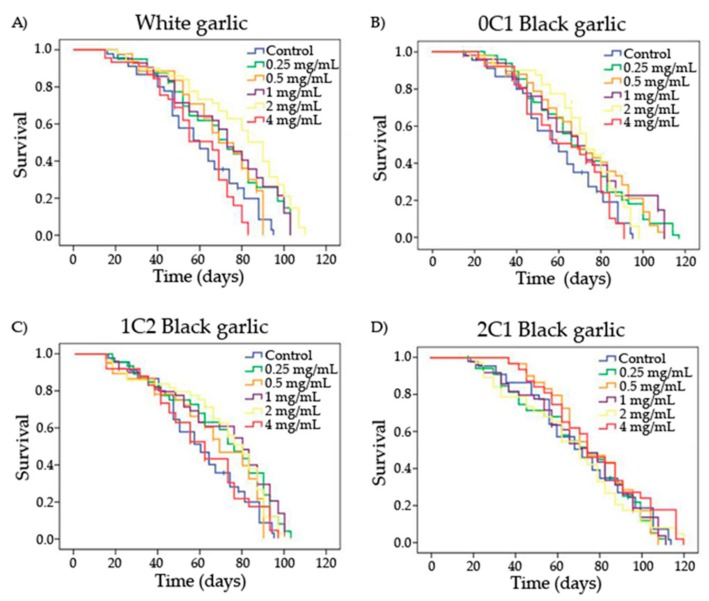
Survival curves of *D. melanogaster* fed with different concentrations of garlic (black and white) over time. **A**) White garlic, **B**) 0C1: black garlic with 13 days aging, **C**) 1C2: black garlic with 32 days aging and **D**) 2C1: black garlic with 45 days aging.

The entire lifespan curves and significances obtained by the Kaplan–Meier method for each substance and concentration are shown in Figure 2 and Table 3, respectively. *Drosophila* had an average lifespan expansion of 60 days in the control treatment. White and 1C2 black garlic significantly increased *Drosophila*’s lifespan at the lowest and the two moderated concentrations tested (0.25, 1, and 2 mg/mL), with an extension with respect to the concurrent control of 10.1, 11.1, and 18.5 days for white garlic and 9.4, 10.1, and 9.8 days for black garlic, respectively (Table 3). Furthermore, every concentration assayed of 0C1 black garlic, except the highest one, induced a lifespan extension of 10 days in *D. melanogaster* compared to the control. On the other hand, 2C1 black garlic did not influence the lifespan extension of *D. melanogaster* at any tested concentration. No previous in vivo studies on longevity activities of black garlic as a food have been reported. However, several authors have reported beneficial effects on animal lifespans using white garlic extracts in *D. melanogaster* at 37.5 and 75 mg/mL, *Caenorhabditis elegans* at 0.05 mg/mL, and senescence-accelerated mice (SAMP8) at 2% (*w/w*) [53,67,68].

We suggest that the differences found between these results and ours could be due to the different types of sample presentation. We used crude entire garlic material, and all data available elsewhere on lifespan trials come from extracts. In this sense, Prowse et al. demonstrated that garlic juice exerted insecticidal activity across life stages of flies at a wide range of concentrations (0.25–5%) in two dipteran pests (*Delia radicum* and *Musca domestica*) [69]. Lei et al. studied the effects of black 10–15 days-aged garlic extracts on the lifespan of *Drosophila* through the observation of half-life time, and the mean and maximum lifespan of organisms. The results suggested a significant longevity extension in *Drosophila* treated with black garlic extracts in a dose-dependent manner [53].

### 3.6. Healthspan Assays 

In order to know the quality of life of the *Drosophila* treated in the longevity assays, we studied the 25% of individual survival at the top of the lifespan curves obtained in the previous test for each substance and concentration tested. This part of the lifespan is considered the healthspan of a curve and is characterized by low and more or less constant age-specific mortality rate values [70]. The results are shown in Table 3.

Only white and 0C1 black garlic induced a significant increase of healthspan in *D. melanogaster* compared to the control, with an average of 8 and 11.5 days, respectively. In contrast, 1C2 and 2C1 black garlic induced a significant reduction of healthspan in *Drosophila* at moderate concentrations, with a value of 7.3 and 9 days, respectively, with respect to the control. No previous studies about the effects that white and black garlic exert on quality of life have been reported.

### 3.7. Cytotoxicity

**Figure 3:**
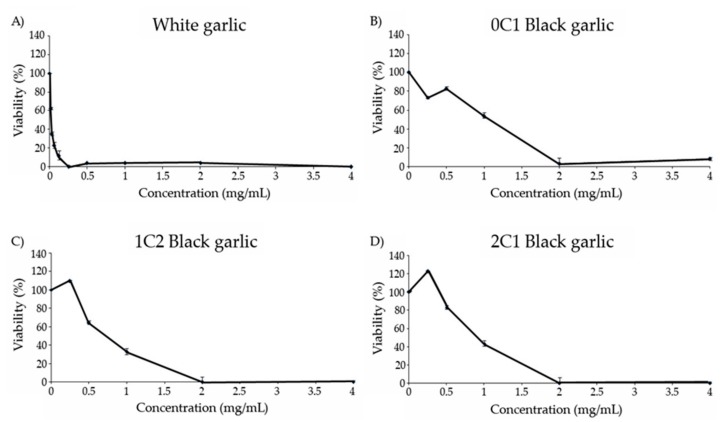
Viability of HL-60 cells treated with different concentrations of black and white garlic for 72 h. (**A**) White garlic, (**B**) 0C1: black garlic with 13 days aging, (**C**) 1C2: black garlic with 32 days aging, and (**D**) 2C1: black garlic with 45 days aging.

All the substances assayed showed cytotoxic activity against HL-60 tumor cells (Figure 3). White and black garlic showed a dose-dependent response, with an increase in the cytotoxicity level as the concentration of garlic increased. White garlic showed the highest cytotoxic effect against the tumor cells, the inhibitory concentration 50 (IC_50_) being under 0.03 mg/mL. 

The cytotoxicity curve of 0C1 black garlic showed a dose-dependent increase with an IC_50_ value equal to 1 mg/mL. In relation to 1C2 and 2C1 black garlic, no inhibition was observed at the lowest concentration tested, but contrarily, a strong tendency to increase cell growth is observed with an IC_50_ value of 0.7 and 0.9 mg/mL, respectively. Moreover, an eventual cell-growth inhibition was observed in 1C2 and 2C1 black garlic at 2 mg/mL. 

A number of studies have demonstrated the chemopreventive activity of garlic by using different garlic preparations, including fresh garlic extract, aged garlic, garlic oil, and a number of organosulfur compounds derived from garlic [71,72]. Such a chemopreventive activity has been attributed to the presence of organosulfur compounds in garlic. Therefore, the consumption of garlic may provide some kind of protection against tumor cell proliferation [73]. Studies on the preventive effects of black garlic extracts also show an induction of in vitro and in vivo inhibition in gastric cancer cell growth, chemopreventive effects in rat colon tumors, and an increase in anti-tumor activity in a mouse model [16,74,75].

### 3.8. DNA Internucleosomal Fragmentation

The HL-60 cell line belongs to the undifferentiated immortal lines, as they are tumor cells. It is widely investigated as a model for purposes of inducible cell differentiation. This phenomenon might affect the cell’s ability to proliferate and therefore their immortality, with the appearance of apoptosis. Compounds capable of inducing differentiation and apoptosis are candidates to act as a chemopreventive agents or cancer chemotherapeutics. 

**Figure 4:**
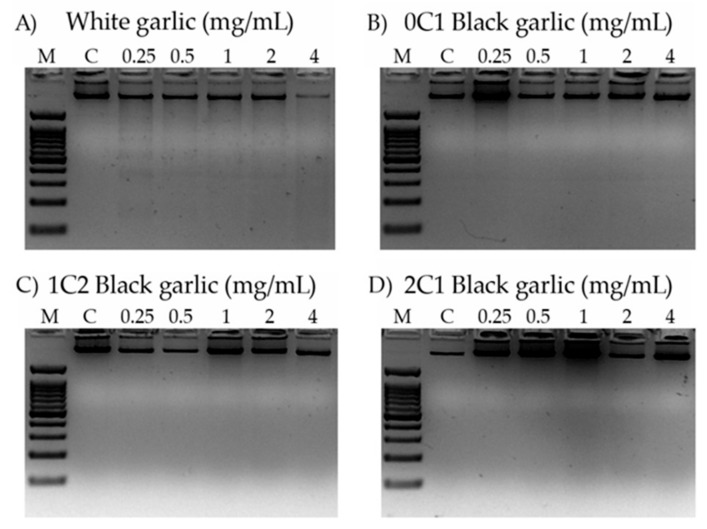
Internucleosomal DNA fragmentation in HL-60 cells treated for 5 h with different concentrations of black and white garlic. DNA fragmentation was detected following electrophoresis in agarose gels and staining with ethidium bromide. M: indicates DNA size marker; C: indicates control (lane 1); 0.25 mg/mL (lane 2); 0.50 mg/mL (lane 3); 1 mg/mL (lane 4); 2 mg/mL (lane 5), and 4 mg/mL (lane 6) of garlic sample. (**A**) White garlic, (**B**) 0C1: black garlic with 13 days aging, (**C**) 1C2: black garlic with 32 days aging, and (**D**) 2C1: black garlic with 45 days aging.

Figure 4 shows the electrophoresis of the genomic DNA of HL-60 cells when treated with different concentrations of white, 0C1, 1C2, and 2C1 black garlic. 

DNA internucleosomal fragmentation is represented by a DNA laddering, and it is associated with the activation of the apoptotic way in cancer cells, a hallmark of the genomic integrity [76]. None of the assayed concentrations (4 mg/mL to 0.25 mg/mL) induced internucleosomal fragmentation by the different black garlic treatments, but a slight fragmentation was observed in the lowest assayed concentration of white garlic (0.25 mg/mL). Hence, the cytotoxic activity observed is only induced in a proapoptotic way in the white garlic.

Our results demonstrate that only white garlic has a strong cytotoxic effect and induces slight DNA proapoptotic internucleosomal fragmentation against HL-60 cells. These results agree with several reports demonstrating that garlic exerts a chemopreventive effect by increasing apoptosis in lung cancer cells (NCI-H1299) [77]. On the other hand, our results do not agree with the results obtained by Wang et al., who detected a dose-dependent apoptosis in aged black garlic extract in in vitro studies [74].

## 4. Conclusions

It is the first time that an investigation of the relationship between the physicochemical characterization and the biological activities of white and black garlic has been carried out. Multifocal studies integrating the toxicity, antitoxicity, genotoxicity, antigenotoxicity, longevity, cytotoxicity, and proapoptotic properties of different types of garlic were followed in order to propose black garlic as a nutraceutical or functional food. 

Black garlic aged for thirteen days showed qualitative improved physicochemical characteristics with respect to white garlic and to the other processed black garlic as well. The 0C1 black garlic (13 days aged) showed similar weight and soluble solids content (°Brix) to the raw garlic. All of the black garlic samples had an improved the polyphenol content and inhibition percentage with respect to the white garlic. 

All types of garlic were safe, not showing toxicity in the *D. melanogaster* model, except for the white one, although only black garlic aged for 13 days showed slight protection against the oxidative toxicant at the three highest concentrations. Genotoxicity assays revealed that all raw and processed garlic were not genotoxic, with the exception of the higher concentration of white garlic, and exhibit moderate antigenotoxic effects when the imaginal discs are treated with the genotoxin hydrogen peroxide. The longevity assays in *D. melanogaster* yielded a significant extension of lifespan results in several concentrations of white and 0C1 and 1C2 black garlic. Finally, the results achieved in the in vitro experiments for garlic cytotoxicity were hopeful. All studied garlic induced a decrease in leukemia cells growth. However, no type of garlic was able to induce proapoptotic internucleosomal DNA fragmentation. 

Important information is added to the agrifood industry as our data suggest that short-aged fermented black garlic (13 days) has higher biological activities than the longer-fermented ones, and even more than white garlic. This could have important industrial and economics consequences. Taking both the physicochemical and biological data, the black garlic aged for 1 days has shown itself to have the best nutraceutical properties. Our findings are relevant for black-garlic-processing agrifood companies as the economical and timing incomes are significantly reduced to 13 days aging.

## Figures and Tables

**Table 1:** Physicochemical characterization of four types of garlic according to the days of aging.

Type of Garlic	White	0C1 Black	1C2 Black	2C1 Black
Aging process (days)	0	13	32	45
Weight of 10 garlic cloves (g)	49.69 ± 0.25 ^a,1^	45.83 ± 0.32 ^b,c^	37.57 ± 0.38 ^b^	19.67 ± 0.19 ^d^
Soluble solid content (ºBrix)	40.47 ± 0.29 ^c^	40.47 ± 0.34 ^c^	43.17 ± 0.48 ^b^	45.67 ± 0.42 ^a^
pH	5.94 ± 0.01 ^a^	3.69 ± 0.03 ^b^	3.60 ± 0.04 ^c^	3.49 ± 0.06 ^d^
Water activity (*a_w_*)	0.97 ± 0 ^a^	0.93 ± 0 ^c^	0.93 ± 0 ^c^	0.93 ± 0 ^c^
Browning intensity (*L*)	47.16 ± 0.15 ^a^	18.73 ± 0.21 ^c^	17.85 ± 0.24 ^b^	17.58 ± 0.25 ^b^
Polyphenol content (g GAE/kg)	4.30 ± 0.04 ^d^	10.94 ± 0.28 ^c^	14.67 ± 0.19 ^b^	16.17 ± 0.29 ^a^
Antioxidant activity (TROLOX equivalents/kg)	10.20 ± 0.27 ^d^	67.65 ± 1.26 ^b^	57.35 ± 1.74 ^c^	78.61 ± 2.41 ^a^

Values are means ± standard error (SE) (*n* = 3). ^1^ Different letters (a, b, c, d) in the same row show significant values in a one-way ANOVA using the post hoc Tukey’s test. GAE, gallic acid equivalents.

**Table 2:** Genotoxicity and antigenotoxicity of white (0 days aging), 0C1 black (13 days aging), 1C2 black (32 days aging), and 2C1 black (45 days aging) garlic in the *Drosophila* wing spot test.

Compound	Clones per Wing (n° spots) ^1^					Inhibition Percentage (%) ^2^
	Number of Wings	Small Single Clones (1–2 Cells) *m* = 2	Large Simple Clones (More Than 2 Cells) *m* = 5	Twin Clones *m* = 5	Total Clones *m* = 2	
H_2_O	41	0.146 (6)	0.049 (2)	0	0.195 (8)	
H_2_O_2_	40	0.350 (14)	0.075 (3)	0	0.425 (17) +	
**Simple Treatment**						
White garlic (mg/mL)						
0.25	40	0.225 (9)	0.025 (1)	0.025 (1)	0.275 (11) −	
2	40	0.375 (15)	0.050 (2)	0.000	0.425 (17) +	
0C1 Black garlic (mg/mL)						
0.25	40	0.175 (7)	0.025 (1)	0.000	0.200 (8) −	
2	41	0.122 (5)	0.000	0.000	0.122 (5) −	
1C2 Black garlic (mg/mL)						
0.25	40	0.200 (8)	0.025 (1)	0.025 (1)	0.250 (10) -	
2	40	0.175 (7)	0.025 (1)	0.000	0.200 (8) -	
2C1 Black garlic (mg/mL)						
0.25	40	0.175 (7)	0.05 (2)	0.000	0.225 (9) −	
2	40	0.250 (10)	0.000	0.000	0.250 (10) −	
**Combined Treatment With H_2_O_2_ (0.12 M)**						
White garlic (mg/mL)						
0.25	34	0.235 (8)	0.088 (3)	0.000	0.323 (11) −	24
2	34	0.265 (10)	0.206 (7)	0.000	0.500 (17) +	−17
0C1 Black garlic (mg/mL)						
0.25	30	0.5 (15)	0.033 (1)	0.000	0.533 (16) +	−25.4
2	30	0.233 (7)	0.033 (1)	0.000	0.266 (8) −	37.4
1C2 Black garlic (mg/mL)						
0.25	26	0.307 (8)	0.038 (1)	0.000	0.346 (9) −	18.6
2	38	0.368 (14)	0.053 (2)	0.000	0.421 (16) −	0.17
2C1 Black garlic (mg/mL)						
0.25	28	0.357 (10)	0.036 (1)	0.000	0.393 (11) −	7.5
2	28	0.357 (10)	0.250 (7)	0.000	0.607 (17) +	−42.8

^1^ Statistical diagnosis according to Frei and Wurgler [29,30]. +, positive (*p* < 0.05); −, negative. m, multiplication factor. Levels of significance α = β = 0.05, tail test without Bonferroni correction. Inconclusive results were resolved by Mann–Whitney–Wilcoxon U-test. ^2^ The inhibition percentages for the combined treatments were calculated from total spots per wing according to Abraham [31].

**Table 3:** Mean and significances of lifespan and healthspan curves for the different garlic treatments assayed in *D. melanogaster*.

Compound Title	Treatment (mg/mL)	Mean Lifespan (Days)		Mean Healthspan (Days)	
Negative Control	0	60.31		32.46	
WG	0.25	70.47	*	38.40	ns
	0.5	68.72	ns	29.40	ns
	1	71.43	**	40.39	*
	2	78.89	***	40.44	*
	4	58.21	ns	31.91	ns
0C1	0.25	70.15	*	37.67	ns
	0.5	71.72	**	38.18	ns
	1	70.73	*	30.85	ns
	2	71.80	*	43.90	*
	4	62.60	ns	33.10	ns
1C2	0.25	69.73	*	29.36	ns
	0.5	65.19	ns	25.15	*
	1	70.49	**	31.10	ns
	2	70.18	*	28.57	ns
	4	60.26	ns	26.20	ns
2C1	0.25	59.06	ns	24.07	ns
	0.5	64.75	ns	41.29	ns
	1	59.82	ns	28.50	ns
	2	57.30	ns	23.46	**
	4	66.38	ns	40.50	ns

Results were calculated by the Kaplan–Meier method, and the significance of the curves was determined by the Log-Rank method (Mantel–Cox). ns: non-significant (*p* > 0.05), *: significant (p < 0.05), **: significant (*p* < 0.01), ***: significant (*p* < 0.001). WG: white garlic (0 days aging); 0C1: black garlic (13 days aging); 1C2: black garlic (32 days aging); 2C1: black garlic (45 days aging).
